# Detection of sputum by interpreting the time-frequency distribution of respiratory sound signal using image processing techniques

**DOI:** 10.1093/bioinformatics/btx652

**Published:** 2017-10-13

**Authors:** Jinglong Niu, Yan Shi, Maolin Cai, Zhixin Cao, Dandan Wang, Zhaozhi Zhang, Xiaohua Douglas Zhang

**Affiliations:** 1 School of Automation Science and Electrical Engineering, Beihang University, Beijing, China; 2 Beijing Engineering Research Center of Diagnosis and Treatment of Respiratory and Critical Care Medicine, Beijing Chaoyang Hospital, Beijing, China; 3 Faculty of Health Sciences, University of Macau, Taipa, Macau, China; 4 The State Key Laboratory of Fluid Power Transmission and Control, Zhejiang University, Hangzhou, China; 5 Department of Statistical Science, Duke University, Durham, NC, USA

## Abstract

**Motivation:**

Sputum in the trachea is hard to expectorate and detect directly for the patients who are unconscious, especially those in Intensive Care Unit. Medical staff should always check the condition of sputum in the trachea. This is time-consuming and the necessary skills are difficult to acquire. Currently, there are few automatic approaches to serve as alternatives to this manual approach.

**Results:**

We develop an automatic approach to diagnose the condition of the sputum. Our approach utilizes a system involving a medical device and quantitative analytic methods. In this approach, the time-frequency distribution of respiratory sound signals, determined from the spectrum, is treated as an image. The sputum detection is performed by interpreting the patterns in the image through the procedure of preprocessing and feature extraction. In this study, 272 respiratory sound samples (145 sputum sound and 127 non-sputum sound samples) are collected from 12 patients. We apply the method of leave-one out cross-validation to the 12 patients to assess the performance of our approach. That is, out of the 12 patients, 11 are randomly selected and their sound samples are used to predict the sound samples in the remaining one patient. The results show that our automatic approach can classify the sputum condition at an accuracy rate of 83.5%.

**Availability and implementation:**

The matlab codes and examples of datasets explored in this work are available at *Bioinformatics* online.

**Supplementary information:**

Supplementary data are available at *Bioinformatics* online.

## 1 Introduction

Sputum is produced by the trachea when the trachea is stimulated. Usually when there is sputum in the trachea, the sputum will be ejected through coughing. However, people who are unconscious, especially patients in Intensive Care Unit (ICU) whose breathing is assisted by a ventilator, cannot eject the sputum by themselves. They need medical staff to check the condition of the sputum and remove the sputum using a suction catheter. If the sputum is not timely removed, it can lead to hypo ventilation, dioxide retention and even pulmonary infection. For this nursing work of clearing the sputum in the trachea, the most important is to determine the sputum condition. The traditional method for this determination involves using lung sounds auscultation to get lung sounds from different parts of the lung. Experienced physicians can determine whether there is sputum in the trachea through distinguishing the sputum sound from mixture sounds. However, this job is time-consuming and requires a skill that is difficult to acquire (Jones *et al.*, 2000; Ranson *et al.*,2014; Sarkar *et al.*, 2015; Yao *et al.*, 2014). Therefore, methods and devices that can accurately detect the condition of the sputum should be investigated. So far, there has been research that focuses on abnormal sound. For example, Habukawa *et al.* (2013) used breath sounds to evaluate the control level of asthma. Pinho *et al.* (2015) adopted fractal dimension and box filtering methods to detect the crackle sounds. Bahoura (2009) used pattern recognition methods to classify normal and wheeze sounds. Abbasi et al. (2013) applied Neural Network and Support Vector Machines to classify normal and abnormal sounds. However, few studies involve sputum detection (Shang, 2011; Oliveira *et al.*, 2013; Paratz, *et al.*, 2014). Usually, electronic stethoscopes were used for data acquisition (Riella *et al.*, 2009; Zolnoori *et al.*, 2012). Some research also used two or more sound sensors to detect the signal. Azarbarzin *et al.* (2011) and Waitman *et al.*(2000) placed two microphones in different parts of the breast to record data. Charleston *et al.* (2011) placed 25 sound sensors on the back of the body to obtain the sound signals. All of the devices used in these studies require physical contact with the bodies of the patients. But this extended physical contact can lead to discomfort. For sputum detection, it is necessary to monitor sputum condition in real time. In this paper, we develop a new automatic approach that can be used to measure respiratory sounds of trachea in real time and analyze those sounds in order to detect the condition of the sputum.

Compared with other respiratory sound measurement devices, the sound sensor of our device is embedded in the ventilation tube near the mouth. This helps prevent the noise from the environment from affecting our measurements when we measure the respiratory sound. Currently, most research just focuses on the waveform of a signal and its mathematical procedures. These methods differ from the image recognition that can give better visualization of sputum conditions. Consequently, it is necessary to prepare visual-based evidence of auscultation sound from a practical viewpoint. In this paper, we propose a method for sputum detection by interpreting the time-frequency distribution of respiratory sound signals using image processing techniques. The time-frequency distribution of respiratory sound signals, determined from the spectrum, is treated as an image. After preprocessing, a gray level co-occurrence matrix is used to extract the texture features. Through extracting sputum-specific features, we can then use machine learning to diagnose the sputum condition.

The aim of our research is to develop an approach that can automatically detect the sputum in real-time and inform the medical staff when sputa exist in the respiratory tract of a patient. Indeed, for an in-house experiment with 272 respiratory sound samples (including 145 sputum and 127 non-sputum), our automatic approach can correctly diagnose the sputum at an accuracy of 83.5%. Moreover, this method can be used practically in clinical environments.

## 2 Materials and methods

**Fig. 1.**
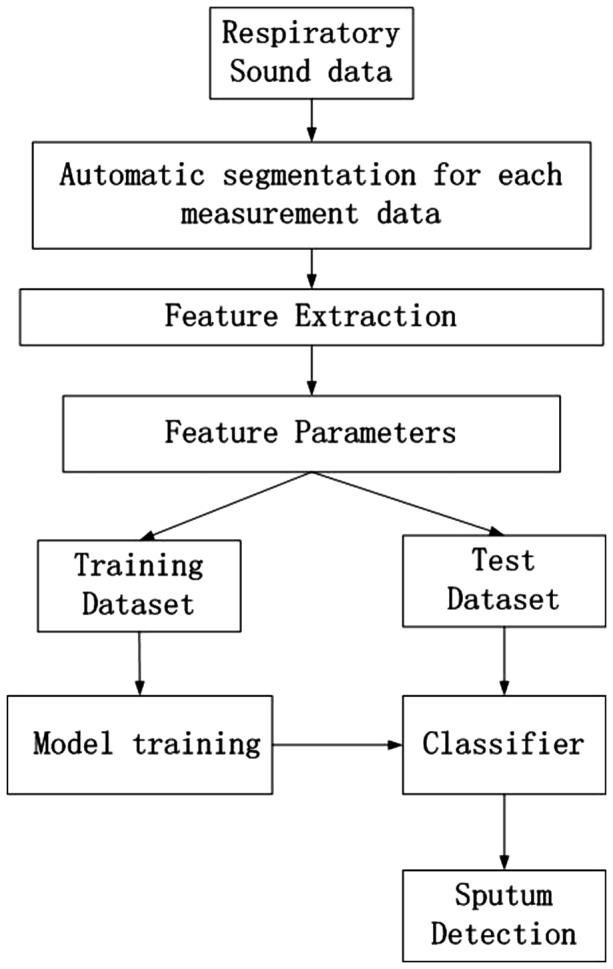
An overview of the method for sputum detection

A device is used to measure the respiratory sound from the trachea. After obtaining the sound data, the method of segmentation is taken to divide them into several segments, each of which includes one respiratory cycle. Each segment consists of several frames. We employ texture of the time-frequency distribution spectrum as features for each sound dataset. Using these features, the segments are classified into sputum sound and non-sputum sound. In the feature extraction step, the Gray Level Co-occurrence Matrices (GLCM) are used. The classifier is then set up for classification. The overview of our proposed method is shown in Figure 1. 

### 2.1 Data acquisition device

**Fig. 2.**
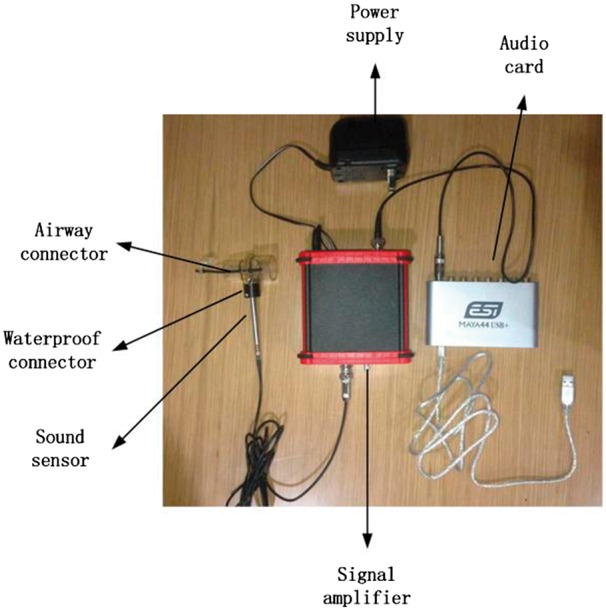
Respiratory sound acquisition. The respiratory sound signal was acquired using the captive microphone with a frequency response from 20 to 20 000 Hz and a dynamic range from 30 to 126

In this study, a new device was developed to measure the respiratory sound signals from the trachea. As shown in Figure 2, this device consists of six major parts: a sound sensor, an audio card, an airway connector, a waterproof connector, a signal amplifier and a power supply. The sound sensor is embedded in the respiratory tube. This way, the noise of environment can be lessened which can increase the signal-to-noise ratio. The amplifier is used to amplify and denoise the sound signal. The waterproof connector is used to keep the head of the sound sensor desiccated. The audio card is used to connect with the computer and transform the analog signal to a digital one so that the sound can be recorded by the computer with a sampling rate of 44 100 HZ. 

### 2.2 Data analysis

**Fig. 3.**
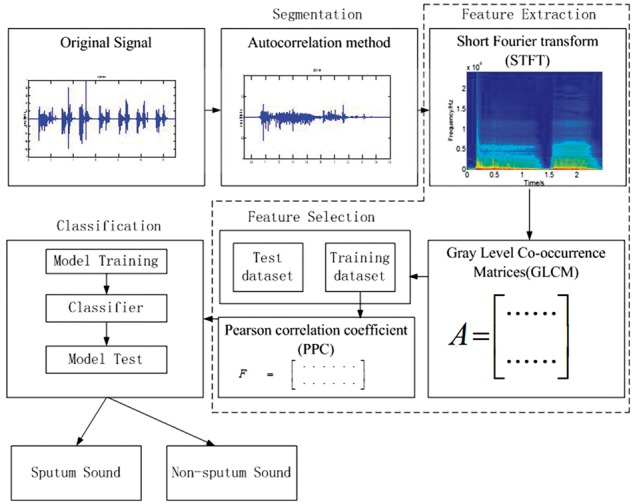
Procedure of data analysis: segmentation, feature extraction, feature selection and classification. Autocorrelation method was used to segment the signal. Feature vectors of signals were obtained by using GLCM and PPC. Logistic classifier was used for classification

In order to use respiratory sound signals to distinguish between sputum and non-sputum, we need to obtain the features inherent in the signals and use these features for classification. The process for data analysis includes segmentation, feature extraction, feature selection, model training and model testing (Fig. 3). Respiratory sound acquisition and feature extraction are performed using code from MatLab (See the Supplementary material for the MatLab codes). Feature selection and classification are performed with the WEKA machine learning tool available at http://www.cs.waikato.ac.nz/ml/weka/. 

#### *2.2.1* Segmentation of input lung sound

An autocorrelation method is carried out in the segmentation step (Jalil *et al.*, 2013). Each recording signal will be separated into many frames by a window function. In this paper, a Hamming window is used. The autocorrelation $R_{i}(x)$ of the $i^{th}$ frame of recording sound signal can be calculated as follows.

(1) $$R_{i}(k)=\sum_{m=0}^{L-1-k}\left[x_{i-1}(i+m)w(m)][x_{i}(i+m+k\right)w(k+m)]$$

where $x_{i}(i+m)$ is the $i^{th}$ (*i = 1,…, M*) frame of the signal. M is the total number of frames. L is the length of the data frame and k is the time shift used to compute the autocorrelation. In the experiment, the values of L and k are 1024 and 400 respectively.

Two thresholds, $C_{1}$ and $C_{2}$, are calculated to segment the sound data. Based on the property of respiratory sound, the $C_{1}$ and $C_{2}$ are calculated by the formula $C_{1}=aC_{0}$ and $C_{2}=bC_{0}$. The values of *a* and *b* should be adjusted based on the measurement environment. The $C_{0}$ is the first silent frames of the recording sound signal. It is assumed that the first 0.025 s of recording sound signal are silent frames, and the average of their maximum of autocorrelated functions $C_{0}$ is calculated. Because the autocorrelation of noise is much lower than the respiratory sound, the maximum of autocorrelation is lower than $C_{1}$. When the maximum of autocorrelation ($\max R_{i}(k)$) is greater than $C_{2}$, it can be used to judge whether the frames of sound belong to the respiratory cycle. And when the maximum value of autocorrelation is greater or less than $C_{1}$, it can be used to judge the start or end of respiratory cycle. In this paper, to prevent the short stop between inspiration and expiration from affecting the segmentation, we introduce the max silence time $T_{s}$ whose value is based on the breathing frequency. The section with low autocorrelation whose lasting time is lower than $T_{s}$ will still be treated as a portion of a respiratory cycle.

#### *2.2.2* Feature extraction

**Fig. 4.**
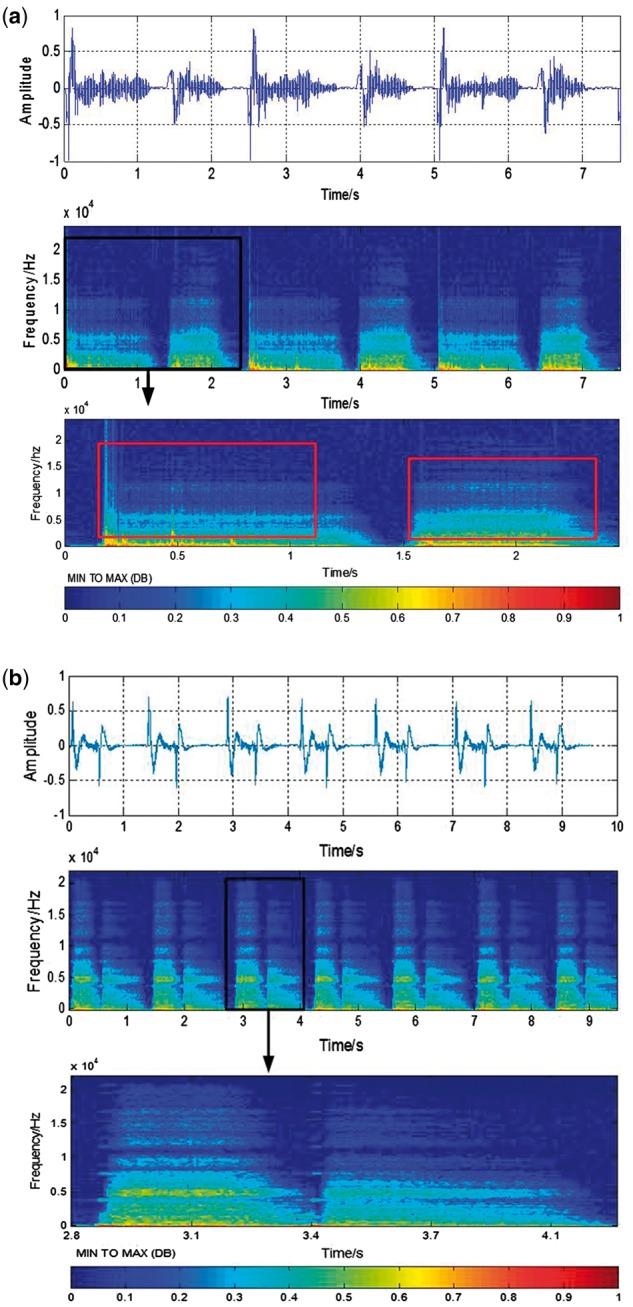
Time-frequency spectrum of signal. Image (**a**) and image (**b**) represent sputum sound signal and non-sputum signal respectively. Both images include three parts, the first part is the original wave signal of respiratory sound. After STFT, the time-frequency distribution of signal is represented by the second part. Then through segmentation, the third part that represents the time-frequency distribution of one respiratory cycle is determined. The texture features are extracted from the image of the time-frequency distribution

From the image of the time-frequency distribution of respiratory sound signals as described in Figure 4, the difference between the time-frequency distribution of sputum and non-sputum sound signals is displayed on the texture of the image. In Figure 4a, some vertical textures can be seen in the red rectangle. However, in Figure 4b, there are few vertical textures. We can use these textures for classification and sputum detection. That is, the time-frequency distribution of respiratory sound signals, determined from the spectrum, is treated as an image, and the sputum detection is performed by interpreting the patterns in the image. Short-time Fourier transform (STFT) is used to get the time-frequency distribution of sound signal (Wang *et al.*, 1993; Yu *et al.*, 2016) and gray level co-occurrence matrices (GLCM) is used to extract the texture parameters from the image of the time-frequency distribution. 

STFT of discrete time signal x(n) can be calculated as:

(2) $$X(n,w_{k})=\sum_{m=0}^{\infty }x(m)*w(n-m)*\;\text{exp}\;[\frac{-2\pi jkm}{N}]$$

where m is the number of frames in advance of the signal, *N* is the length of the frame in advance of the signal and the value of N is 1024. w(n) is the window function. In this study, Blackman–Harris window is chosen. Computing the result of this equation with a digital signal and specific window function yields a matrix of complex numbers. It can be expressed as followed:

(3) $$X(n,k)=\sum_{m=0}^{N-1}x_{n}(m)e^{-j\frac{2\pi km}{N}}$$

To get a digital spectrogram from this, the magnitude of each number in the STFT matrix is computed and squared.

(4) $$E(n,k)={\left|X(n,k)\right|}^{2}=(X(n,k))\times (conj(X(n,k)))$$

where E(n,k) is the power spectrum and its value can present the image gray level, *n* is treated as the horizontal axis and *k* is treated as the vertical axis. The DB spectrum image is then calculated by using a transformation of $10\;{\text{log}}_{10}(E(n,k))$. Using the method above, we determine the time-frequency spectrum as shown in Figure 4.

After getting the image of time-frequency distribution, the GLCM is used to quantitatively evaluate textural parameters (Mohanaiah *et al.*, 2013; Soh *et al.*, 1999; Zucker *et al.*,1980). The GLCM is a matrix where the number of rows and columns is equal to the number of gray level *G* in the image. The matrix element P(i,j|d,ψ) is the relative frequency of two pixels which are separated by a pixel distance $d=\sqrt{\Delta x^{2}+\Delta y^{2}}$ in the direction of ψ. The element P(i,j|d,ψ) contains the second order statistical probability and can be written as

(5) P(i,j,d,ψ)={(x,y),(x+Δx,y+Δy)|f(x,y)=i;f(x+Δx,y+Δy)=j}

Due to their large dimensionality, the GLCM is very sensitive to the size of the texture samples on which they are estimated. Thus, the number of gray levels is often reduced. Prior to matrix calculation, the input gray level of image was reduced to 16 levels while maintaining the histogram shape. Four important texture features, angular second moment (energy), inertia moment, correlation and entropy, were selected.

Angular Second Moment (Energy)

(6) $$Energy=\sum_{i=1}^{g}\sum_{j=1}^{g}P^{2}(i,j,d,\psi )$$

Inertia moment

(7) $$IN=\sum_{i=1}^{g}\sum_{j=1}^{g}\left[{\left(i-j\right)}^{2}P^{2}(i,j,d,\psi \right)]$$

Correlation

(8) $$Correlation=\sum_{i=1}^{g}\sum_{j=1}^{g}[i\times j\times P(i,j,d,\psi )-u_{1}\times u_{2}]/(d_{1}\times d_{2})$$

where

$$u_{1}=\sum_{i=1}^{g}i\sum_{j=1}^{g}P(i,j,d,\psi )\;u_{2}=\sum_{j=1}^{g}j\sum_{i=1}^{g}P(i,j,d,\psi )$$

$$d_{1}^{2}=\sum_{i=1}^{g}{\left(i-u_{1}\right)}^{2}\sum_{j=1}^{g}P(i,j,d,\psi )\;d_{2}^{2}=\sum_{j=1}^{g}{\left(j-u_{1}\right)}^{2}\sum_{i=1}^{g}P(i,j,d,\psi )$$

Entropy

(9) $$Entropy=-\sum_{i=1}^{g}\sum_{j=1}^{g}P(i,j,d,\psi )\times {\text{log}}_{10}P(i,j,d,\psi )$$

Based on the data we collected and similar respiratory sound analysis (Nogata *et al.*, 2015), the most texture concentrates on four directions (ψ = 0°, 45°, 90°, 135°). Therefore, each feature measure is obtained for 4 angles (ψ = 0°, 45°, 90°, 135°). We get 4 × 4 = 16 attributes.

#### *2.2.3* Classification method

Before the classification step, relevant and descriptive features should be selected. A good set of this feature is the one that contains features highly correlated with class. In other words, if the feature is correlated with the class, it will be useful in classification (Hall, 1999). The Pearson correlation coefficient (PCC) gives an indication of the strength of the linear relationship between features and result of classification. Based on the value of PCC, the useful feature can be selected (Zhou *et al.*, 2016). Thus, to find the optimal feature set, a feature selection method based on PCC is used in this paper.

The task of classifying is deciding class membership y’ of an unknown item x’ based on a dataset $D=(x_{1},y_{1}),\cdot \cdot \cdot (x_{n},y_{n})$ for item *x_i_* with known class memberships $y_{i}$. In this study, the type of classification is a dichotomous classification. The class labels are either sputum sound or non-sputum sound. $y_{i}=0$ represents non-sputum sound and $y_{i}=1$ represents sputum sound. $x_{i}$’s are usually *d*-dimensional vectors which are attributes of the signal. Currently, logistic regression is a popular method to model binary data and is often used in biomedical data processing (Press *et al.*, 1978; Subasi and Erçelebi, 2005). Therefore, logistic regression is used to classify the sound data. To avoid unstable parameter estimation when the number of covariates is relatively large or when the covariates are highly correlated, the logistic regression model with a ridge estimator is applied (Lee *et al.*, 1988).

Suppose that there are n observations $\left(X_{i},Y_{i}\right)$, where Y is defined as $Y_{i}=1$ for a sputum sound signal and $Y_{i}=0$ otherwise, $X_{i}$ are *d*-dimensional feature row vectors of the signal. Then the probability that *Y_i_=1*, given the value of $X_{i}=(X_{i1},\cdot \cdot \cdot X_{i16})$, is denoted by $p(X_{i})$ and is modeled with the standard logistic regression model.

(10) $$p(X_{i})=\frac{1}{1+e^{-g(X_{i},\theta_{i})}}$$

where $g(X_{i},\theta )=\theta_{0}+\theta_{1}X_{i1}+\theta_{2}X_{i2}+\cdot \cdot \cdot +\theta_{16}X_{i16}$, in this paper, θ is a 17-dimensional vector of parameters which can be regarded as weight of each feature parameter. The probability of $Y_{i}$ is determined by the value of θ. The vector θ is chosen so that it maximizes $p(X_{i})$. Based on the $p(X_{i})$, the classification result can be acquired. There is no constant term involved in this regression problem. The log-likelihood *l* of data (*X*, *Y*) under this model is

(11) $$l(\theta )=\sum_{i}\left[Y_{i}\;\text{log}\;p(X_{i})+(1-Y_{i})\;\text{log}\;(1-p(X_{i}))\right]$$

When there is correlation between the various explanatory variables, it will lead to unstable parameters estimates. If θ can be shrunk towards 0 and allow a little bias, it can stabilize the system and provide more appropriate estimates. Therefore the log-likelihood l(θ) can be rewritten as follows:

(12) $$l^{\lambda }(\theta )=l(\theta )-\lambda \left\|\theta \right\|$$

where l(θ) is the unrestricted log-likelihood function and $\left\|\theta \right\|={\left(\sum \theta_{j}^{2}\right)}^{1/2}$ is the norm of the parameter θ.λ is the ridge parameter that is used to shrink the norm of θ. When λ=0, the problem will become maximum likelihood estimation. With a small value of λ, a little bias will be introduced to the system, which will stabilize the system and lower the variance of the estimation. The value of ${\hat{\theta }}^{\lambda }$ can be obtained when the equation reaches its maximum. This way, the estimate ${\hat{\theta }}^{\lambda }$ will be on average closer to the real value of θ.

The ${\hat{\theta }}^{\lambda }$ can be obtained by the Quasi-Newton method (Richard *et al.*,1989). The first derivative of $l^{\lambda }(\theta )$ is

(13) $$\nabla l^{\lambda }(\theta )=\sum_{i}X_{i}(Y_{i}-p(X_{i}))-2\lambda \theta$$

The first step is to construct an objective function. This step is analogous to Taylor‘s transformation.

(14) $$m(s)=l^{\lambda }({\hat{\theta }}_{k}^{\lambda })+\nabla l^{\lambda }{\left({\hat{\theta }}_{k}^{\lambda }\right)}^{T}s+\frac{s^{T}Bs}{2}$$

where $l^{\lambda }({\hat{\theta }}_{k}^{\lambda }+s)\approx m(s)$. *B* is an approximation to the Hessian matrix which is a positive definite matrix. The gradient of equation (14) is

(15) $$\nabla l^{\lambda }({\hat{\theta }}_{k}^{\lambda }+s)\approx \nabla l^{\lambda }({\hat{\theta }}_{k}^{\lambda })+Bs$$

To get the optimal solution, *B* is chosen to satisfy the Equation (14) and the gradient is set to zero. Define $s=s_{k}$, $B=B_{k}$, Therefore 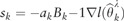, where $a_{k}=\kappa a_{k-1}(0<\kappa <1)$ should satisfy the Armijo-Goldstein rule condition that is used to ensure a sufficient descent (Renato *et al.*,1984). Set ${\hat{\theta }}_{k+1}^{\lambda }={\hat{\theta }}_{k}^{\lambda }+s_{k}$, where $s_{k}$ is the searching direction to ensure that the iteration is working. Equation (16) can be identified as

(16) $$B_{k}({\hat{\theta }}_{k+1}^{\lambda }-{\hat{\theta }}_{k}^{\lambda })=\nabla l({\hat{\theta }}_{k+1}^{\lambda })-\nabla l({\hat{\theta }}_{k}^{\lambda })$$

**Fig. 5.**
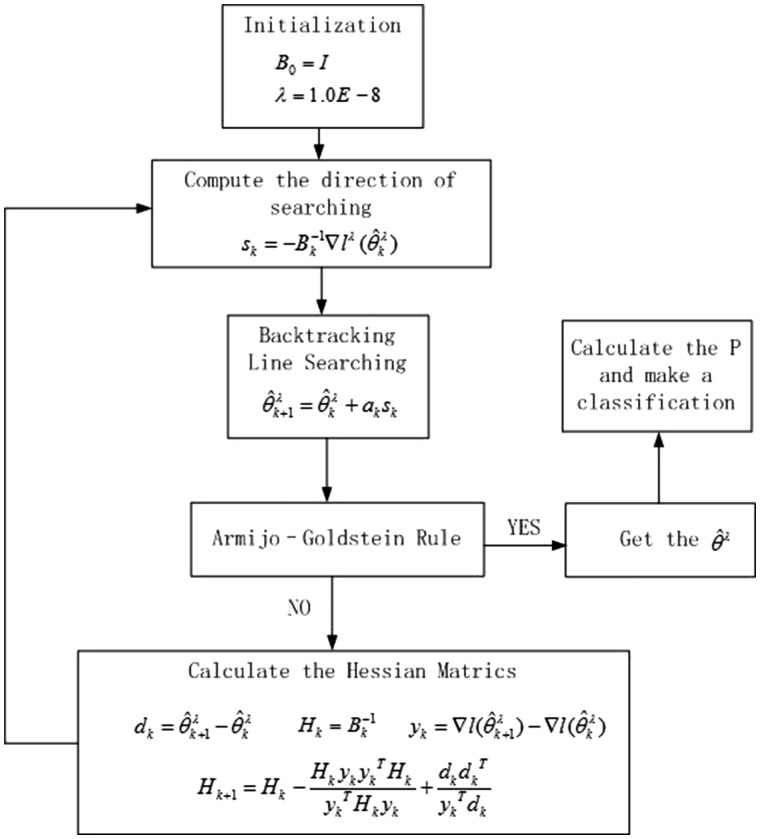
The block diagram for the logistic algorithm

When *B_k_* satisfies Equation (16), it can be used to update ${\hat{\theta }}_{k}^{\lambda }$ to be ${\hat{\theta }}_{k+1}^{\lambda }$. In this paper, the DFP method (Richard *et al.*, 1989) is used to calculate *B_k_*. Suppose that $d_{k}={\hat{\theta }}_{k+1}^{\lambda }-{\hat{\theta }}_{k}^{\lambda }$, $y_{k}=\nabla l({\hat{\theta }}_{k+1}^{\lambda })-\nabla l({\hat{\theta }}_{k}^{\lambda })$, $H_{k}=B_{k}^{-1}$. The iteration formula of $B_{k}$ can be written as follows: (17)
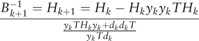
 With the iteration, the estimate ${\hat{\theta }}^{\lambda }$ can be calculated. In this paper, $B_{0}$ and λ are set as $B_{0}=I$, $\lambda =1.0\times 10^{-8}$, respectively. Figure 5 shows the iteration steps for calculating $p(X_{i})$ in formula (10) and the class of classification can be determined based on the value of $p(X_{i})$. 

## 3 Results

**Fig. 6.**
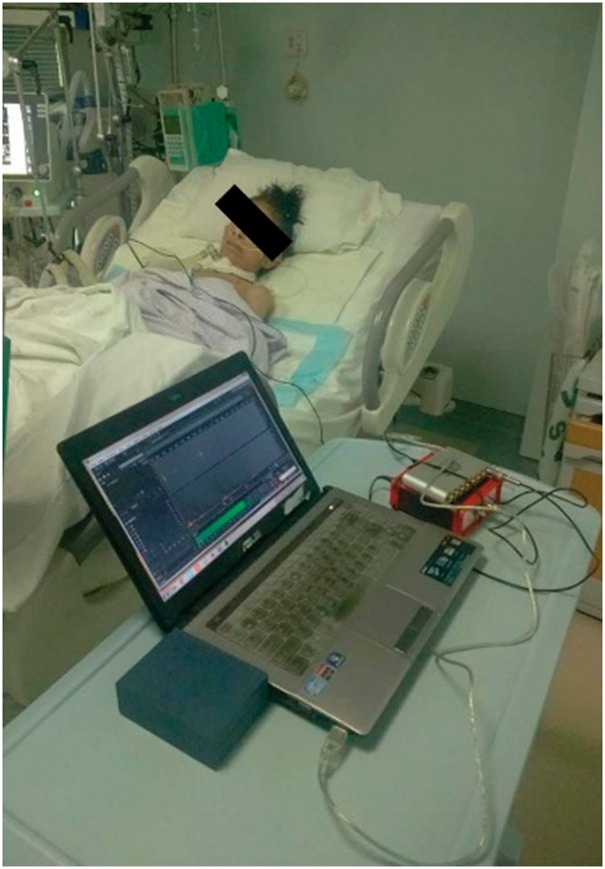
Measurement environment. The device is connected to the tube of ventilator near the mouth of a patient. The respiratory sounds are measured by the sound sensor and then transformed into a digital signal by the audio card at a sampling rate of 44 100 Hz

We conducted an experiment to test our proposed approach. In this experiment, 272 respiratory sound samples were collected from 12 intubated patients (with 145 being sputum sounds and 127 being non-sputum sounds) in the ICU of Chaoyang Hospital. The age of the patients ranged from 60 to 85 years old. Because certain lung diseases and other diseases (such as pneumonia, respiratory failure, cerebellum infarctions, etc.) may lead to the lack of ability in breathing by themselves, they were intubated to help them breath. The measurement environment is shown in Figure 6. Before the experiment, the waterproof connector and other equipment was sterilized with autoclave. 

Based on the result of the suction and the judgement of experienced nurses, the presence of sputum was determined and categorized as either sputum or non-sputum. When there was secretion after suction, the sounds recorded in the 1–2 min before suction began will be treated as the sputum sound. The sound in 1–2 min recorded after suction was completed was treated as non-sputum sound. The accuracy in detection of the presence of sputum was then evaluated.

### 3.1 Training data and test data

**Fig. 7.**
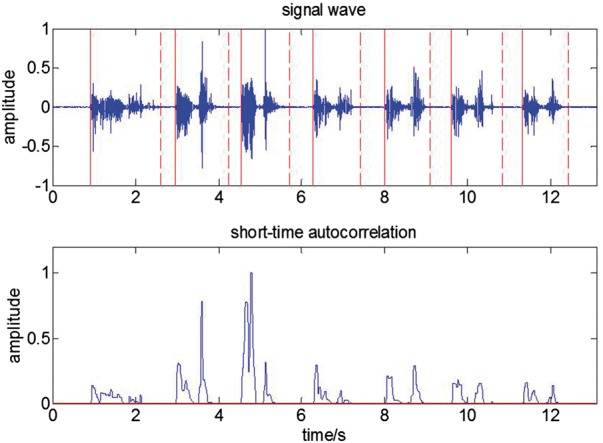
Segmentation. The signal wave was segmented by the red solid lines and red dashed lines. The red solid line is the start of the respiratory cycle and the red dash line is the end of the cycle. The bottom panel shows short-time autocorrelation

The data before the suction was defined as sputum sound and those after suction was regarded as non-sputum sound. The respiratory sound consists of one or two minute waveforms. All the data was automatically segmented by the autocorrelation method. Through the simulation using Matlab, as shown in Figure 7, the red solid line is the start of a respiratory sound cycle and the red dashed line represents the end of a respiratory sound cycle. 

To evaluate the performance of classifiers, we set the training and test sets using the cross-validation strategy applied to the patients as follows. Among the 12 patients, we select one patient and all the sound samples from this patient to form a test set. The sound samples from the remaining 11 patients form the training set. For each classifier, we use the training dataset to predict the test set and obtain a corresponding discrimination rate. We then apply this process to each of the 12 patients. The overall performance is the average of the 12 values of discrimination rate for each classifier. A total of 272 sound samples (145 sputum sounds and 127 non-sputum sounds) from 12 patients were tested in this cross-validation process. For each classifier, we look at the highest discrimination rate and its corresponding number of features.

In this paper, to minimize the effect of the correlations among samples from the same patients on the classification, two methods are taken. One is to use the data that comes from the different measurement time (each patient will be measured 2–4 times.). Because the sputum is related to the patient situation that is easily affected by the treatment performance (Burgel *et al.*, 2009), it is always changing. The generation of sputum sound is more like a random procedure. The correlation of data comes from different times are treated as uncorrelated. The other is to define the training data and test data from the different patients so that the test set comes from a patient that is independent from the patients in the training set.

### 3.2 Analytic result

Our proposed method can be evaluated by discrimination rate: how many segments were correctly classified and how large the sensitivity and specificity of the diagnosis were. The sensitivity is the proportion of actual positives that are correctly identified whereas specificity is the proportion of negatives that are correctly identified.

$$Sensitivity=\frac{TP}{TP+FN}$$

$$Specificity=\frac{TN}{TN+FP}$$

where TP, TN, FP and FN denote true-positive, true-negative, false-positive and false-negative, respectively. In order to compare the proposed method with the other classification method, Bayesnet, Naïve Bayes, K-nearest neighbors (KNN), RandomForest and Reptree were also tested.

To test the reliability of the segmentation method described in 2.2.1, we apply the method to 239 segment samples (including 111 sputum and 128 non-sputum segments) all with known positions. The accuracy of segmentation is 98.7% overall, 98.2% for the sputum segments and 99.2% for the non-sputum segments, which indicates that the segmentation method is reliable.

As described in Section 2.2.3, PCC was used to rank an attribute. The results are shown in Table 1. The highest value of PCC was energy in a 45-degree direction and the lowest value was entropy in a 45-degree direction (Table 1).

**Table 1.** Correlation between attribute and sputum status

Rank|	Feature	Direction	Coefficient
1	Energy	45	0.130
2	Inertia	90	0.130
3	Energy	135	0.125
4	Energy	0	0.125
5	Energy	90	0.122
6	Inertia	135	0.111
7	Inertia	0	0.110
8	Correlation	45	0.089
9	Correlation	0	0.088
10	Correlation	135	0.088
11	Correlation	90	0.087
12	Entropy	90	0.032
13	Entropy	135	0.031
14	Entropy	0	0.031
15	Inertia	45	0.019
16	Entropy	45	0.018

**Fig. 8.**
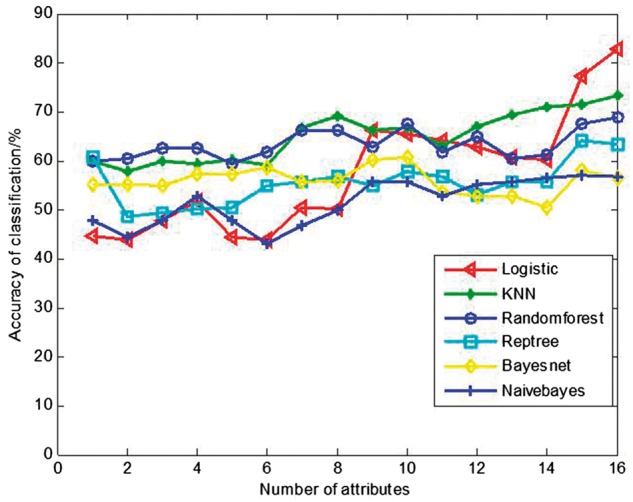
The accuracy of classification with various classifiers

In order to compare the logistic model with other commonly used existing classification methods, Bayesnet, Naïve Bayes, KNN, RandomForest and Reptree were also tested. Following the rank order in Table 1, different numbers of features were chosen to conduct discrimination using the cross-validation process described in Section 3.1 for each classifier. The results are shown in Figure 8, which indicate that the highest discrimination rate is achieved using the logistic regression model with all the 16 attributes. 

The highest discrimination rate and its corresponding number of attributes for each classifier are shown in Table 2, which leads to two discoveries. First, the three classifiers with the single highest discrimination rates, i.e. Logistic, KNN and Random forest, all achieved their highest discrimination rates when all the 16 attributes were included in the model, indicating that all the 16 attributes make contribution to classification and should be included in the classification analysis. Second, among the 6 tested classifiers, the logistic model achieves the highest discrimination rate of 83.5%. Based on these discoveries, we adopted the logistic model for discriminating sputum sounds in our automatic sputum detection procedure. The sensitivity and specificity of this adopted classifier is further shown in Table 3, which indicates that the sensitivity and specificity are 82.1 and 85.0%, respectively.

**Table 2.** The highest discrimination rate and its corresponding number of features for each classifier

Classifier	Highest discrimination rate (%)	Number of attributes
Logistic	83.5	16
KNN	73.2	16
Random forest	68.7	16
Reptree	64.0	15
Bayesnet	60.0	10
Naïve Bayes	57.1	15

**Table 3.** Confusion matrix for the logistic method

Signal	Class		Accuracy (%)
	Sputum	Non-sputum	
Sputum	119	26	82.1
Non-sputum	19	108	85.0

## 4 Discussion

Several techniques for sound feature extraction in either the time, frequency or complex domains have been developed (Azarbarzin *et al.*, 2009; Charleston *et al.*,2011; Riella *et al.*, 2009; Waitman *et al.*, 2000; Zolnoori *et al.*, 2012). However, most of them (Azarbarzin *et al.*, 2009; Charleston *et al.*,2011; Waitman *et al.*, 2000; Zolnoori *et al.*, 2012) have focused on the extraction of the information contained in only a narrow band of the signal spectrum, centering on the fundamental or one of the harmonics of the respiratory sound signal frequency. In this paper, the method of features extraction in the joint time-frequency domain was proposed. The spectrum gives a one to one mapping between each signal component. There are no interference terms. The pattern can be displayed in the distribution and correctly reflect the local energy distribution over the time and frequency domains. Based on the GLCM method for texture feature extraction, the wave signal recognition was transformed into a visual-based recognition. This constitutes a novel wide band analysis technique. In addition, to reduce heavy background noise, the sound sensor was embedded in a thick tube which was used to connect the trachea with the airway of the ventilator.

There are five steps in the process of classification. They are respiratory data acquisition, auto-segmentation, feature extraction, feature selection and classification. Because the sound sensor was embedded into respiratory airway, the thick tube of airway can minimize the background environment noise. Because of this, the signal-to-noise ratio can be increased. Auto-segmentation was performed by the maximum of autocorrelation function. Based on the property of respiratory sound, the threshold of $C_{1}$ and $C_{2}$ are calculated by the formulas $C_{1}=aC_{0}$ and $C_{2}=bC_{0}$ respectively. The segmentation method is reliable with an accuracy of over 98%.

In this paper, various classifiers (including the logistic model, Bayesnet, Naïve Bayes, KNN, RandomForest and Reptree) were investigated. Table 2 shows that the highest accuracy was obtained by using the logistic model and it can reach 83.5%. The sputum sound and non-sputum sound were 82.1 and 85.0% respectively (Table 3). The classification accuracy of non-sputum sound is higher than the sputum sound.

In this study, experiments were conducted using real data recorded at the ICU. Our method achieved a discrimination rate of about 83% which can be accepted by the doctor in ICU. The experimental results show that the proposed detection system is able to effectively detect respiratory sounds associated with sputum for the real environments in the hospital. Our proposed approach is a necessary and novel way to help doctors or nurses judge when to proceed with endotracheal suctioning and reduce the frequency of unnecessary suction. According to American Association for Respiratory Care (AARC) guideline (AARC, 1993), the endotracheal suctioning should be performed at a minimum frequency or when clinically indicated (i.e. when complications due to accumulated secretions are manifested). Since endotracheal suctioning can cause hypoxemia, mechanical trauma, bronchospasm and hemodynamic instability, an accurate assessment of the need for suctioning may decrease the frequency of suctioning complications.

For future work, possible improvements can be introduced by increasing the number of features, implementing recently developed non-linear metrics such as sample entropy (Zhang *et al.*, 2017) and strictly standardized mean difference (Zhang *et al.*, 2011) and selecting more appropriate training samples (Ren *et al*., 2017). In addition, more subjects with various types of sounds will be tested. In clinical practice, the first question to be asked is whether there exists a sputum condition. Thus, in this paper, we develop an automatic approach to detect whether a sputum condition exists. In addition to answering the question of whether a sputum condition exists, doctors are also interested in predicting the amount, proportion and location of sputum. Hence, there is also a future need to build models capable of detecting that information.

## Funding

This work was supported by the National Natural Science Foundation of China (Grant No. 51575020), Open Foundation of the State Key Laboratory of Fluid Power and Mechatronic Systems and the Start-up Research Grant (SRG2016-00083-FHS) at University of Macau.

*Conflict of Interest*: none declared.

## Supplementary Material

Supplementary Data
