# Supplementary material for: Detection of sputum by interpreting the time-frequency distribution of respiratory sound signal using image processing techniques
Source: Bioinformatics. 2017 Oct 13;34(5):820–7. doi: 10.1093/bioinformatics/btx652 (PMC6192228; doi:10.1093/bioinformatics/btx652)
Supplement: Supplementary Data [file btx652_supp.zip › btx652-suppl_data/supplementary material.docx]

Supplementary Material

Detection of Sputum by Interpreting the Time-frequency Distribution of Respiratory Sound Signal Using Image Processing Techniques

Jinglong NIU^1,2, +^, Yan SHI^1,2,3, +,*^, Maolin CAI^1,2^ , Zhixin CAO^2^, Dandan WANG^3^, Zhaozhi ZHANG^4^ and Xiaohua Douglas ZHANG ^3,*^

^1^School of Automation Science and Electrical Engineering, Beihang University, Beijing 100191, CHINA., ^2^ Beijing Engineering Research Center of Diagnosis and Treatment of Respiratory and Critical Care Medicine, Beijing Chaoyang Hospital, Beijing 100043, China, ^3^ Faculty of Health Sciences, University of Macau, Taipa, Macau, ^4^ Department of Statistical Science, Duke University, Durham, NC27708, USA.

The codes should be run in the matlab software. In Part 1, the codes are used to conduct the segmentation of sounds. In Part 2, the codes are used to extract texture features from the image that comes from the Short-time Fourier Transformation. After running the codes, 4 feature parameters will be obtained. They are energy, inertia moment, correlation and entropy. Each feature is obtained for 4 angles (θ=0︒,45︒,90︒,135︒). Therefore, 4×4=16 attributes can be acquired. In the supplementary materials, there are two files whose name are “sputum_sound_sample” and “nonsputum_sound_sample”. The format of samples are .xlsx. These two files contain the sound sample data that can be used to test the codes. The sputum sound data is in the “sputum_sound_sample” file. The non-sputum sound data is in the “nonsputum_sound_sample” file. After running the codes below, the features can be obtained.

Data processing steps of running codes

1. Load the data into Matlab software. This processing can be conducted by the codes as follows:

If the storage format of data is .wav, please run the codes: “[ss,fs,bits]=wavread(file); xx=ss(:,1);” If the storage format of data is .xlsx, please run the codes: “[xx]=xlsread(file.xlsx); fs=44100, bits=32;”

1. Run the code in the part1 to conduct segmentation.

These codes can be divided into three parts- preparation for the segmentation, endpoint detection and segment acquisition. Preparation for the segmentation is used to split the signal into frames and calculate the thresholds. The part of endpoint detection is used to detect the endpoint of each segment. Finally, the segment data can be acquired using the codes in the third part.

1. After acquiring the segment data of respiratory sound, run the code in part2 to acquire the texture features of signal.

In this step, codes can also be divided into three parts-STFT transformation, image matrix acquisition and the feature vector calculation. The time-frequency spectrum can be acquired through the STFT transformation. Then the spectrum is transformed into gray image and gray level is reduced to 16. Finally, the texture features of image can be acquired using the GLMC code.

The flow chart of running codes is shown as the flows:


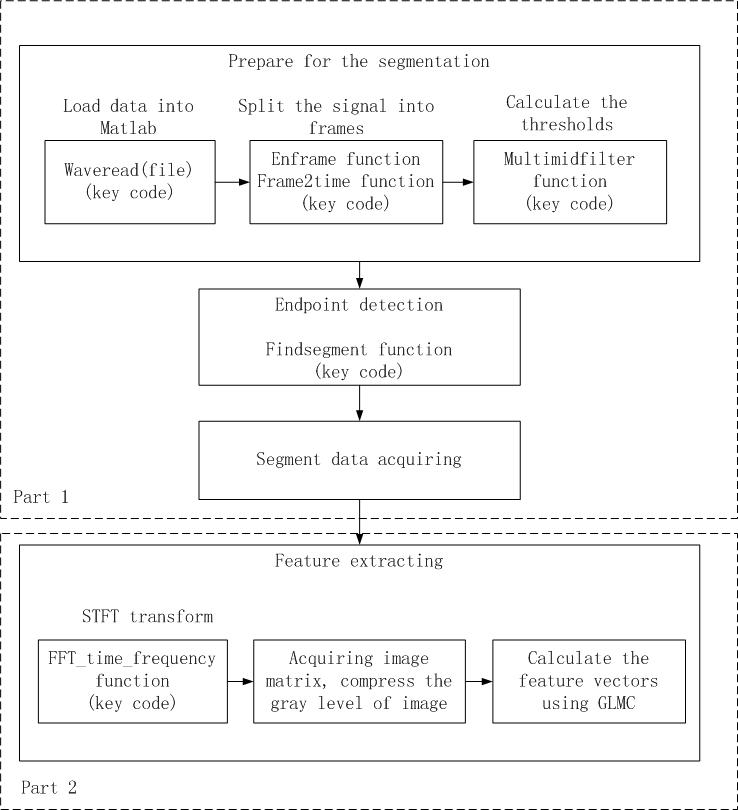


**Part1 Code for segmentation**

------------------ **Prepare for the segmentation** ---------------------

[ss,fs,bits]=wavread(file); % load the signal

xx=ss(:,1);

% because in the supplementary the data’s format is .xlsx, if you want to use this data, please replace the code above with the code “[xx]=xlsread(file.xlsx); fs=44100, bits=32;”

xx=xx-mean(xx); %

x=xx/max(abs(xx)); %

N=length(x); %

time=(0:N-1)/fs;

signal=x; %

wlen=1024; inc=400; % wlen is the length of window inc is the shifting time

IS=0.25; the first 0.025s recording sound

wnd=hamming(wlen); %

overlap=wlen-inc; %

NIS=fix((IS*fs-wlen)/inc +1); % calculate the silent frames in the head of signal

y=enframe(signal,wnd,inc)'; % frame processing

fn=size(y,2); %

frameTime=frame2time(fn, wlen, inc, fs);% calculate each frame's time

for k=2 : fn %

u=y(:,k);

ru=xcorr(u);

Ru(k)=max(ru);

end

Rum=multimidfilter(Ru,10); % smooth processing

Rum=Rum/max(Rum); % normalization

thredth=max(Rum(1:NIS)); % calculate threshold

T1=1.5*thredth;

T2=30*thredth;

----------------------------------------------------------------

**enframe, frame2time,** **multimidfilter function applied by above code**

**---------------------------------enframe function--------------------------**

function [f,t]=enframe(x,win,inc)

nx=length(x(:));

nwin=length(win);

if (nwin == 1)

len = win;

else

len = nwin;

end

if (nargin < 3)

inc = len;

end

nf = fix((nx-len+inc)/inc);

f=zeros(nf,len);

indf= inc*(0:(nf-1)).';

inds = (1:len);

f(:) = x(indf(:,ones(1,len))+inds(ones(nf,1),:));

if (nwin > 1)

w = win(:)';

f = f .* w(ones(nf,1),:);

end

if nargout>1

t=(1+len)/2+indf;

end

----------------------------------------------------------------

**-----------------------------frame2time function------------------------------**

function frameTime=frame2time(frameNum,framelen,inc,fs)

frameTime=(((1:frameNum)-1)*inc+framelen/2)/fs;

----------------------------------------------------------------**------------------------------multimidfilter function-------------------------**

function y=multimidfilter(x,m)

a=x;

for k=1 : m

b=medfilt1(a, 5);

a=b;

end

y=b;

----------------------------------------------------------------

**---------------------------------Endpoint detection--------------------------**

function [voiceseg,vsl,SF,NF]=endpoint_detection(dst1,T1,T2)

%input:

% dst1:signal T1: First threshold T2: the Second threshold

% output:

% voiceseg: information about the result of segmentation vs1: the

% the numbers of segments

% SF=1: the frames of respiratory sound NF=0: the frames of non-respiratory sound

fn=size(dst1,2); % get the frame

maxsilence = 10; % initialize

minlen = 10;

status = 0;

count = 0;

silence = 0;

%start endpoint detection

xn=1;

for n=2:fn

switch status

case {0,1} % 0 = silent, 1 =start

if dst1(n) > T2 %

x1(xn) = max(n-count(xn)-1,1);

status = 2;

silence(xn) = 0;

count(xn) = count(xn) + 1;

elseif dst1(n) > T1 % silent

% zcr(n) < zcr2

status = 1;

count(xn) = count(xn) + 1;

else %

status = 0;

count(xn) = 0;

x1(xn)=0;

x2(xn)=0;

end

case 2, % voice

if dst1(n) > T1 %

count(xn) = count(xn) + 1;

else %

silence(xn) = silence(xn)+1;

if silence(xn) < maxsilence %

count(xn) = count(xn) + 1;

elseif count(xn) < minlen %

status = 0;

silence(xn) = 0;

count(xn) = 0;

else %

status = 3;

x2(xn)=x1(xn)+count(xn);

end

end

case 3, % voice over and prepare for the next part of voice

status = 0;

xn=xn+1;

count(xn) = 0;

silence(xn)=0;

x1(xn)=0;

x2(xn)=0;

end

end

el=length(x1);

if x1(el)==0, el=el-1; end %

if el==0, return; end

if x2(el)==0 %

%fprintf('Error: Not find endding point!\n');

x2(el)=fn;

end

SF=zeros(1,fn); %

NF=ones(1,fn);

for i=1 : el

SF(x1(i):x2(i))=1;

NF(x1(i):x2(i))=0;

end

speechIndex=find(SF==1); % calculate voiceseg

voiceseg=findSegment(speechIndex);

vsl=length(voiceseg);

----------------------------------------------------------------

**----------------------------findSegment function------------------------------**

function soundSegment=findSegment(express)

if express(1)==0

voicedIndex=find(express); % find the 1 in the express

else

voicedIndex=express;

end

soundSegment = [];

k = 1;

soundSegment(k).begin = voicedIndex(1); % set the start place of the first segment

for i=1:length(voicedIndex)-1,

if voicedIndex(i+1)-voicedIndex(i)>1, % the end of this segment

soundSegment(k).end = voicedIndex(i); % set the end place of the segment

soundSegment(k+1).begin = voicedIndex(i+1);% set the start place of the next segment

k = k+1;

end

end

soundSegment(k).end = voicedIndex(end); %

% calculate the length of segment

for i=1 :k

soundSegment(i).duration=soundSegment(i).end-soundSegment(i).begin+1;

end

----------------------------------------------------------------

**-------------------------------Acquire the segment data----------------------**

[voiceseg,vsl,SF,NF]=endpoint_detection(Rum,T1,T2)

for k=1 : vsl %

nx1=voiceseg(k).begin; nx2=voiceseg(k).end;

nx3=voiceseg(k).duration;

time_length(k)=nx3;

j=1;

for n=fix(frameTime(nx1)*fs):fix(frameTime(nx2)*fs)

segment(j)=xx(n);

j=j+1;

end

s=['(storage path)\sound_segments',int2str(k),'.wav'];

wavwrite(segment,fs,bits,s);

clear segment;

end

**Part2 Code for feature extraction**

**------------------------main program(feature extraction)---------------------**

clear all;

folder = 'storage path of segments of sound\';

files = dir([folder '*.wav']);

for i=1:length(files)

file = [folder files(i).name];

res(i).file = file;

try

[y,Fs,bits] = wavread(file);

res(i).y = y;

res(i).Fs = Fs;

res(i).bits = bits;

catch

warning( ['reading ' file ' ERROR']);

end

end

for i_file=1:length(files)

wav_data=res(i_file).y;

Fs=res(i_file).Fs ;

Features(i_file,:)=Feature_extraction(wav_data,Fs);

end

**-----------------------------------------------------------------------------**

**-----------------Code for feature Extraction---------------------------------**

(1)function [feature_result]=Feature_extraction(wav_data,Fs) % Get the 16 features for each segmentation of signal

% mid_result matrix: 16 features

% wave_data: segment data

%Fs: the frequency of sigal

Winsiz=1024; Shift=2; Base=-70; Mode=1; Gray=32; %

[x,y,Fig]=FFT_time_frequency(Winsiz,Shift,Base,Mode,Gray,wav_data,Fs );

[mm nn]=size(Fig);

Fig1=Fig(1:mm/3,1:nn);

length1=256; length2=2048;

II=im2uint8(imresize(Fig1, [length1,length2]));

img=II;

imgn=img;

%--------------------------------------------------------------------------

%Gray Level co-occurrence matrix

%--------------------------------------------------------------------------

Gray=imgn;

[M N]=size(Gray);

%--------------------------------------------------------------------------

%Compress the level of gray to 16

%--------------------------------------------------------------------------

count_g=256/16;

for i = 1:M

for j = 1:N

for n = 1:count_g/1

if (n-1)*16<=Gray(i,j)&Gray(i,j)<=(n-1)*16+15

Gray(i,j) = n-1;

end

end

end

end

% figure

% imshow( Gray);

%--------------------------------------------------------------------------

% In this part four matrix will be calculated, angle is 0,45,90,135

%--------------------------------------------------------------------------

P = zeros(count_g,count_g,4);

for m = 1:count_g

for n = 1:count_g

for i = 1:M

for j = 1:N

if j<N&Gray(i,j)==m-1&Gray(i,j+1)==n-1

P(m,n,1) = P(m,n,1)+1;

P(n,m,1) = P(m,n,1);

end

if i>1&j<N&Gray(i,j)==m-1&Gray(i-1,j+1)==n-1

P(m,n,2) = P(m,n,2)+1;

P(n,m,2) = P(m,n,2);

end

if i<M&Gray(i,j)==m-1&Gray(i+1,j)==n-1

P(m,n,3) = P(m,n,3)+1;

P(n,m,3) = P(m,n,3);

end

if i<M&j<N&Gray(i,j)==m-1&Gray(i+1,j+1)==n-1

P(m,n,4) = P(m,n,4)+1;

P(n,m,4) = P(m,n,4);

end

end

end

if m==n

P(m,n,:) = P(m,n,:)*2;

end

end

end

%%---------------------------------------------------------

% Normalization

%%---------------------------------------------------------

for n = 1:4

P(:,:,n) = P(:,:,n)/sum(sum(P(:,:,n)));

end

%--------------------------------------------------------------------------

%Calculate matrix to get parameters energy entropy inertance and

%correlation

%--------------------------------------------------------------------------

H = zeros(1,4);

I = H;

Ux = H; Uy = H;

deltaX= H; deltaY = H;

C =H;

for n = 1:4

E(n) = sum(sum(P(:,:,n).^2));

for i = 1:count_g

for j = 1:count_g

if P(i,j,n)~=0

H(n) = -P(i,j,n)*log(P(i,j,n))+H(n);

end

I(n) = (i-j)^2*P(i,j,n)+I(n);

Ux(n) = i*P(i,j,n)+Ux(n);

Uy(n) = j*P(i,j,n)+Uy(n);

end

end

end

for n = 1:4

for i = 1:count_g

for j = 1:count_g

deltaX(n) = (i-Ux(n))^2*P(i,j,n)+deltaX(n);

deltaY(n) = (j-Uy(n))^2*P(i,j,n)+deltaY(n);

C(n) = i*j*P(i,j,n)+C(n);

end

end

C(n) = (C(n)-Ux(n)*Uy(n))/deltaX(n)/deltaY(n); %correlation

end

%-----------------------------------------------------------------------

%get the value of energy, entropy, moment of inertia, standard deviation

%------------------------------------------------------------------------

direction_num=4; feature_num=4;

feature_sum=direction_num*feature_num;

for i=1:direction_num

result_mid(1,i)=E(i);

result_mid(2,i)=H(i);

result_mid(3,i)=I(i);

result_mid(4,i)=C(i);

end

result_mid(:,[1,3])=result_mid(:,[3,1]);

result_mid=result_mid';

count_sum=1;

for i=1:feature_num

for j=1:direction_num

feature_result(count_sum)=result_mid(i,j);

count_sum=count_sum+1;

end

end

-----------------------------------------------------------------------------

**----------------------------FFT_time_frequency-------------------------------**

function [x_time,y_frequent,Fig]=FFT_time_frequency(Winsiz,Shift,Base,Mode,Gray,wav_data1,Fs)

wav_data=wav_data1(:,1);

% x=resample(wav_data,16000,Fs);

% Fs=16000;

%[x,Fs]=wavread('npu.wav');

x=wav_data;

n=fix((length(x)-Winsiz)/Shift)+1;

A=zeros(1+Winsiz/2,n);

%s=enframe(x,Winsiz,Shift);

for i=1:n

n1=(i-1)*Shift+1;

n2=n1+(Winsiz-1);

s=x(n1:n2);

s=s.*blackmanharris(Winsiz);

z=fft(s);

%z=fft(s(i,:));

z=z(1:(Winsiz/2)+1);

z=z.*conj(z);

z=10*log10(z);

A(:,i)=z;

end

L0=(A>Base);

L1=(A<Base);

B=A.*L0 +Base*L1;

L=(B- Base)./(max(max(B))- Base);

y=[0:Winsiz/2]*Fs/Winsiz;

x=[0:n-1]*Shift;

if Mode==1

colormap('jet' );

else

mymode =gray;

mymode =mymode (Gray: - 1:1,:);

colormap(mymode);

end

Fig=L; y_frequent=y; x_time=x;

-----------------------------------------------------------------------------
